# Molecular Understanding of Growth Inhibitory Effect from Irradiated to Bystander Tumor Cells in Mouse Fibrosarcoma Tumor Model

**DOI:** 10.1371/journal.pone.0161662

**Published:** 2016-08-25

**Authors:** Sejal Desai, Nishad Srambikkal, Hansa D. Yadav, Neena Shetake, Murali M. S. Balla, Amit Kumar, Pritha Ray, Anu Ghosh, B. N. Pandey

**Affiliations:** 1 Radiation Biology and Health Sciences Division, Bhabha Atomic Research Centre, Mumbai, Maharashtra, India; 2 Advanced Centre for Training, Research and Education of Cancer, Tata Memorial Centre, Kharghar, Navi Mumbai, Maharashtra, India; 3 Homi Bhabha National Institute, Mumbai, Maharashtra, India; Center for Cancer Research, UNITED STATES

## Abstract

Even though bystander effects pertaining to radiation risk assessment has been extensively studied, the molecular players of radiation induced bystander effect (RIBE) in the context of cancer radiotherapy are poorly known. In this regard, the present study is aimed to investigate the effect of irradiated tumor cells on the bystander counterparts in mouse fibrosarcoma (WEHI 164 cells) tumor model. Mice co-implanted with WEHI 164 cells γ-irradiated with a lethal dose of 15 Gy and unirradiated (bystander) WEHI 164 cells showed inhibited tumor growth, which was measured in terms of tumor volume and Luc^+^WEHI 164 cells based bioluminescence *in vivo* imaging. Histopathological analysis and other assays revealed decreased mitotic index, increased apoptosis and senescence in these tumor tissues. In addition, poor angiogenesis was observed in these tumor tissues, which was further confirmed by fluorescence imaging of tumor vascularisation and CD31 expression by immuno-histochemistry. Interestingly, the growth inhibitory bystander effect was exerted more prominently by soluble factors obtained from the irradiated tumor cells than the cellular fraction. Cytokine profiling of the supernatants obtained from the irradiated tumor cells showed increased levels of VEGF, Rantes, PDGF, GMCSF and IL-2 and decreased levels of IL-6 and SCF. Comparative proteomic analysis of the supernatants from the irradiated tumor cells showed differential expression of total 24 protein spots (21 up- and 3 down-regulated) when compared with the supernatant from the unirradiated control cells. The proteins which showed substantially higher level in the supernatant from the irradiated cells included diphosphate kinase B, heat shock cognate, annexin A1, angiopoietin-2, actin (cytoplasmic 1/2) and stress induced phosphoprotein 1. However, the levels of proteins like annexin A2, protein S100 A4 and cofilin was found to be lower in this supernatant. In conclusion, our results provided deeper insight about the damaging RIBE in an *in vivo* tumor model, which may have significant implication in improvement of cancer radiotherapy.

## Introduction

Radiotherapy is one of the common modalities for the treatment of cancer patients. However, there are issues such as radio-resistance, recurrence, side effects associated with radiotherapy which pose serious challenge before the clinicians. These issues can be better addressed through deeper insight of radiobiological processes (like bystander effect, genomic instability) under clinical conditions. There are ample situations arise during cancer radiotherapy in which irradiated tumor cells interact with bystander tumor cells. Such interaction known as radiation induced bystander effect (RIBE) may significantly contribute towards clinical outcome of cancer radiotherapy depending on the nature and magnitude of the effect [1–3]. However, molecular understanding of RIBE in relevance to cancer radiotherapy is poorly known.

Expanding body of research has demonstrated RIBE in mammalian cells grown *in vitro* using various biological endpoints like apoptosis, micronuclei formation, mutations, altered gene expression, genomic instability etc [4–7]. Conditioned media transfer [8, 9], microbeam [10] and tissue culture inserts [11] have been commonly used to demonstrate RIBE in various cancer cell lines pertaining to cancer radiotherapy. Although these experimental approaches have provided significant understanding about signaling mechanisms and kinetics of RIBE, they do not accurately represent the physiological conditions and multi-cellular tumor environment [12]. Multi-cellular tissue models like mouse ear model [13] three-dimensional skin [14] trout skin [15] and fish explant [16] have been used to investigate RIBE. However, these studies are mainly related to RIBE associated with radiation risk. *In vivo* RIBE studies pertaining to cancer radiotherapy are rather limited in literature. Xue *et al* [17] demonstrated effect of pre-labeled tumor cells with lethal concentration of ^125^I, on the growth of bystander tumor cells. Recently, use of synchrotron radiation in RIBE studies associated with cancer radiotherapy has been discussed [18]. This warrants the development of approaches to investigate RIBE in *in vivo* systems which are more relevant to cancer radiotherapy.

In the present work, RIBE was studied using a murine allograft tumor model, wherein the ability of irradiated tumor cells (exposed to a lethal dose of gamma radiation *ex vivo*) to elicit radiobiological response in surrounding unirradiated (bystander) tumor cells was investigated. Tumor growth was measured to determine the response of lethally irradiated tumor cells on the bystander tumor cells co-implanted in mice (S1 Fig). Any enhancement or retardation in overall tumor growth would provide a line of evidence in support of *in vivo* bystander effect. We found that the lethally irradiated tumor cells inhibited the growth of tumor formed by bystander cells by inducing apoptosis, senescence and anti-angiogenic mechanisms. These growth inhibitory effects were mediated by soluble factors secreted from the irradiated cells. Putative mediators involved in the observed RIBE were identified using differential proteomics and cytokine profiling of the supernatant.

## Materials and Methods

### Animals

Six–eight weeks old female BALB/c mice were obtained from Bhabha Atomic Research Centre (BARC) animal breeding facility. Mice (5 per cage) were housed in a pathogen-free animal facility with free access to standard mouse chow and water. Animal care and handling followed the protocol approved by BARC Animal Ethics Committee. During the course of study, animals were daily monitored by trained technicians regarding their well being. There was no death of animals due to natural cause or any disease. Animals were euthanized (in a carbon dioxide gas chamber) after completion of experiment or before the tumor size reached ~1800 mm^3^, whichever was earlier. The number of animals used in different experimental groups has been mentioned in the respective figure legends.

### Tumor implantation and tumor volume measurement

A fibrosarcoma cell line (WEHI 164) syngenic to BALB/c mice was obtained from National Institute for Cell Sciences, Pune, India. WEHI 164 cells were maintained in Dulbecco’s Modified Eagle’s Medium (DMEM) supplemented with 10% fetal calf serum and antibiotics (100 units/ml penicillin and 100 μg/ml streptomycin) at 37°C in 5% CO_2_ atmosphere. Cells were maintained in exponentially growing culture conditions and passaged thrice a week. Confluent cultures (~90%) were trypsinised and cells were resuspended in PBS to obtain 1x10^6^ cells/50 μl. The cell suspension was gamma-irradiated with a dose of 15 Gy at room temperature using Cobalt-60 source blood-irradiator (Board of Radiation and Isotope Technology, Mumbai, India; dose rate: 1 Gy/min). After 15 min of irradiation, a cell suspension of control (unirradiated) cells (1x10^6^/50 μl) was mixed with either 50 μl of PBS (control) or with 50 μl of irradiated (15 Gy) tumor cell suspension (1x10^6^ cells). Either control or mixture of cells was injected subcutaneously into the hind leg of two separate groups of mice. In both the cases, total injection volume was 100 μl. Unless stated, ‘mixed cells’ are henceforth referred for the mixture of 1x10^6^ control (unirradiated) and 1x10^6^ irradiated (15 Gy) tumor cells (in suspension total 2x10^6^ cells) and tumor derived from the mixture of these cells are referred as ‘mixed cells tumors’. Tumors derived from 1x10^6^ unirradiated control cells are referred as ‘control tumors’. A schematic outline for the above mentioned experimental approach has been shown in S1 Fig. Viability of control and irradiated cells was evaluated by trypan blue assay, which was found to be ~95% in irradiated tumor cells (15 Gy; after 15 min of irradiation) compared to control cells viability >98%. Wherever required, supernatants from control and irradiated cell suspensions were collected by centrifugation (400 x g; 10 min; room temperature).

Tumor growth was examined by volume measurement which commenced once the tumor reached palpable size on the 7^th^ day post-implantation. Tumors were measured in two perpendicular diameters (large and small) using digital vernier caliper (Nichiryo, Japan). Tumor volume was calculated using the formula V = 0.523A^2^B, where, V: volume in mm^3^, A: larger diameter and B: smaller diameter. The volume of normal leg (at the same position, where tumor volume was measured) in the same mice was subtracted from the tumor volume to obtain the final tumor size. Unless stated, all the assays with the tumor tissues were performed on the tumor samples obtained after 11^th^ day of implantation.

### Development of luciferase expressing WEHI 164 cells for *in vivo* imaging

The WEHI 164 cells were transfected with CMV-FL2-eGFP-pcDNA 3.1(+) plasmid vector by lipofection method [19]. The transfected clones were selected using G418 selection medium. The luminescence of clones was measured in a luminometer at 550 nm and relative luminescence unit (RLU) per μg protein was calculated. Clone expressing highest RLU per μg protein was further cultured in 100 mm dish and maintained in selection medium containing G418 (200 μg/ml of media). These cells were referred as ‘Luc^+^WEHI 164 cells’ and non-transfected cells as ‘WEHI 164 cells’.

Ten minutes prior to imaging, 3 mg of sodium salt of D-luciferin (30 mg/ml stock prepared in sterile PBS; Biosynth, USA) was injected into the peritoneal cavity of mice. After 5 min, mice were anesthetized (isofurane 5% and carbogen 1 liter/min) for 3 min and placed under anesthesia condition (2% isofurane and carbogen 0.5 liter/min) in the imaging chamber of *in vivo* imaging system (Photon Imager, Biospace, France). Images were acquired (after total 10 min of D-luciferin injection) for integration time of 8 min under bioluminescence mode. Bright field reference images of the animals were obtained under low light illumination from light emitting diodes built into the imaging dark box. The intensity of bioluminescence signal was represented as a pseudo-colour image where blue is the least intense and red represents the highest intensity. The two images (bioluminescence and bright field) were superimposed using the in-built software of the instrument. The bioluminescence signals (photons/sec/cm^2^/Sr) representing the luciferase activity in live cells was quantified using data analysis software (M^3^ vision) of the imaging system.

### Histopathology

Sections from the paraffin-embedded tumor samples were stained with hematoxylin-eosin to evaluate tumor growth pattern, morphology, extent of leukocytes infiltration and mitotic rates. The average number of spindle or round shaped cells and mitotic cells/field was calculated from the 10 randomly selected microscopic fields at 40X. The cells which were showing chromatin condensations and/or fragmentations and cytoplasmic shrinkage were characterized as apoptotic. The results were expressed in a semi-quantitative manner using scores based on severity grades ranging from 0 to +3.

### Senescence assay

Senescence associated β-galactosidase activity was assessed in tumor sections using cellular senescence assay kit (Millipore) as per the instructions provided by the manufacturer. In brief, the paraffin embedded tissue sections mounted on the slides were first dewaxed by immersing the slides in xylene followed by bathing the slides in decreasing percentage of ethanol solution for rehydration. The sections were then incubated with X-gal, a chromogenic substrate of β-galactosidase. After overnight incubation, slides were washed with PBS. Then the sections were covered with coverslips and observed under light microscope. β-Galactosidase positive senescent cells appeared blue in colour.

### TUNEL assay

To examine the degree of apoptosis in tumor samples, terminal deoxynucleotidyl transferase dUTP nick end labeling (TUNEL) assay was performed on the tumor sections using in situ cell death TUNEL kit (Roche). In brief, the paraffin embedded tissue sections mounted on the slides were dewaxed followed by rehydration as mentioned in the previous section. The sections were then permeabilized with 0.1% triton X-100 and 0.1% sodium citrate. The sections were overlaid with reaction mixture containing terminal deoxynucleotidyl enzyme and FITC-labelled dNTPs followed by incubation at 37°C for 1h in dark humidified chamber. Finally, slides were washed with PBS, mounted by cover slips with anti-fade mounting media and observed under fluorescent microscope (Eclipse Ti, Nikon, Japan).

### Angiogenesis assay in mice tumors

To determine the magnitude of angiogenesis in control tumors and mixed cells tumors, on the 11^th^ day of implantation, animals were intravenously injected with AngioSense 750 EX (Perkin Elmer, USA; 2 nmol/100 μl prepared in sterile PBS). Fluorescence imaging (excitation: 745 nm; emission: 800 nm; high pass filter cut off: 770 nm; illumination: 30%) was performed 24h after injection of the fluorescence dye in the anesthetized animals (as mentioned previously in ‘In vivo imaging’ section) using animal imaging system (Photon Imager, Biospace, France). Hair was removed from the dorsal side of the animals including the tumor regions to minimize background auto-fluorescence. To determine the expression of CD31, tumor tissues obtained from these mice were fixed in formalin and processed for immuno-histochemistry [mouse anti-CD31 (Cell Signalling Technologies, USA; dilution 1:50) and secondary antibody conjugated with AlexaFluor 488 (goat anti-Rabbit IgG; Invitrogen, USA; dilution 1:200)] [20]. The images were acquired using a fluorescence microscope (Eclipse Ti, Nikon, Japan).

### Cytokine profiling of the supernatants from irradiated and control WEHI 164 cells

Cytokine profile of the supernatants from the control and irradiated cells (15 Gy; 15 min post irradiation obtained by centrifugation 400 x g; 10 min; room temperature) was studied using mouse cytokine panel of enzyme-linked immunosorbent assay (ELISA) array (Signosis, USA) as per the instructions provided by the manufacturer. IL-2 concentration in the supernatant was determined using ELISA kit (BD Biosciences, USA) following protocol provided along with kit by the manufacturer. The absorbance was taken at 450 nm using 96 well plate reader (Infinite, M-200 PRO, Tecan, Switzerland) and analyte concentration was calculated from the standard curve.

### Proteomic analysis of cell supernatants

For comparative proteomic analysis, the supernatants were collected from unirradiated (control) and irradiated (15 Gy) tumor cells through centrifugation as mentioned above. Two dimensional gel electrophoresis (2-DE) and matrix-assisted laser desorptionionization-time of flight mass spectrometry (MALDI-TOF MS) was carried out as per the protocol published earlier [21]. The first dimension isoelectric focusing (IEF) was performed on 17 cm gradient immobilized pH gradient (IPG) strips (pH 3–10 and pH 5–8) on the Protean IEF cell (Bio-Rad, CA, USA). Briefly, the IPG strips were rehydrated with pre-estimated protein samples in 300 μl of rehydration buffer (7M urea, 20mM dithiothreitol (DTT), 4% 3-[(3-cholamidopropyl)-dimethylammonio]-1-propanesulfonate 0.2% carrier ampholytes, 0.0002% bromophenol blue). IEF was performed using a three step program: 250V for 20 min, 10,000V for 4h, and 60,000 V-h. The focused strips were first equilibrated in buffer I [6M urea, 2% SDS, 0.05M tris-Cl (pH 6.8), 20% glycerol and 2% DTT] followed by in buffer II (containing 2.5% iodoacetamide instead of DTT compared to buffer I). All the chemicals used for 2-DE were procured from Bio-Rad, CA, USA.

The second dimensional electrophoresis was performed on 12% SDS PAGE gels using PROTEAN-II (Bio-Rad, CA, USA) vertical gel electrophoresis system at a constant voltage of 85V. After electrophoresis, gels were stained with coomassie blue R-250 and scanned at 350 dots per inch using Image Scanner III (GE Healthcare, USA). The image analysis were performed using PDQuest software (version 8, Bio-Rad, CA, USA) to identify protein spots that showed differential expression at ≥1.5-fold as previously described [21]. Each sample was prepared twice and run on 2-DE in duplicate. Only those protein spots which showed modulation in both the experimental sets were taken for further analysis.For MALDI-TOF-MS analysis, protein spots were manually excised. In-gel digestion was performed with trypsin (25ng/μl in 25mM NH_4_HCO_3_) at 37°C. The resulting tryptic fragments were serially extracted with 0.1% trifluoroacetic acid, 0.1% trifluoroacetic acid in 50% acetonitrile and only acetonitrile. Peptide mass fingerprints of tryptic digests were acquired with MALDI-TOF mass spectrometer (UltraflexII Bruker Daltonics, Germany) operated in positive-ion reflector mode using ά-cyano-4-hydroxycinnamic acid as matrix. Further confirmation of identified proteins was performed by acquiring MS/MS spectra. Protein identification was done using Mascot search engine (http://www.matrixscience.com) by searching in SWISS-PROT database. The search parameters were set as one missed cleavage, error tolerance of ±100 ppm for PMF [except for cofilin1 (50ppm) and Protein CIP2A (120 ppm)] and ± 0.5 Da for MS/MS ion search.

### Statistical analysis

Unless mentioned, results are expressed as mean ± standard error of mean (SEM) of three independent experiments. Statistical analysis was performed using two-tailed Student's ‘t’ test using Origin 8 software. Any comparison was considered statistically significant at p <0.05.

## Results

### Effect of irradiated cells on the tumor growth of bystander tumor cells

To study the effect of irradiated tumor cells on the growth of bystander tumor cells under *in vivo* conditions, mice were implanted with irradiated tumor cells mixed with bystander tumor cells and the tumor growth was monitored. However, in this approach, tumor cells irradiated with sub-lethal *ex vivo* doses of radiation would survive along with the bystander cells and thus, contribute in the tumor formation. This may result in ambiguity in the interpretation of the results. Hence, to ensure that the tumor formed will be only from the bystander cells, it was necessary to determine the lethal or non-tumorigenic radiation dose. For this, WEHI 164 cells (1x10^6^), irradiated with different doses (5–15 Gy) were implanted sub-cutaneously in the hind leg of the animals. Tumor growth was monitored 7^th^ day onwards. Compared to control, tumors produced from 5 Gy and 10 Gy irradiated cells showed slower rate of tumor growth whereas 15 Gy irradiated cells could not form the tumor (S2 Fig). Animals implanted with 15 Gy irradiated tumor cells didn’t show tumor when followed upto 30 days after implantation (data not shown). This suggests 15 Gy as non-tumorigenic dose and thus was used for further studies.

**Fig 1:**
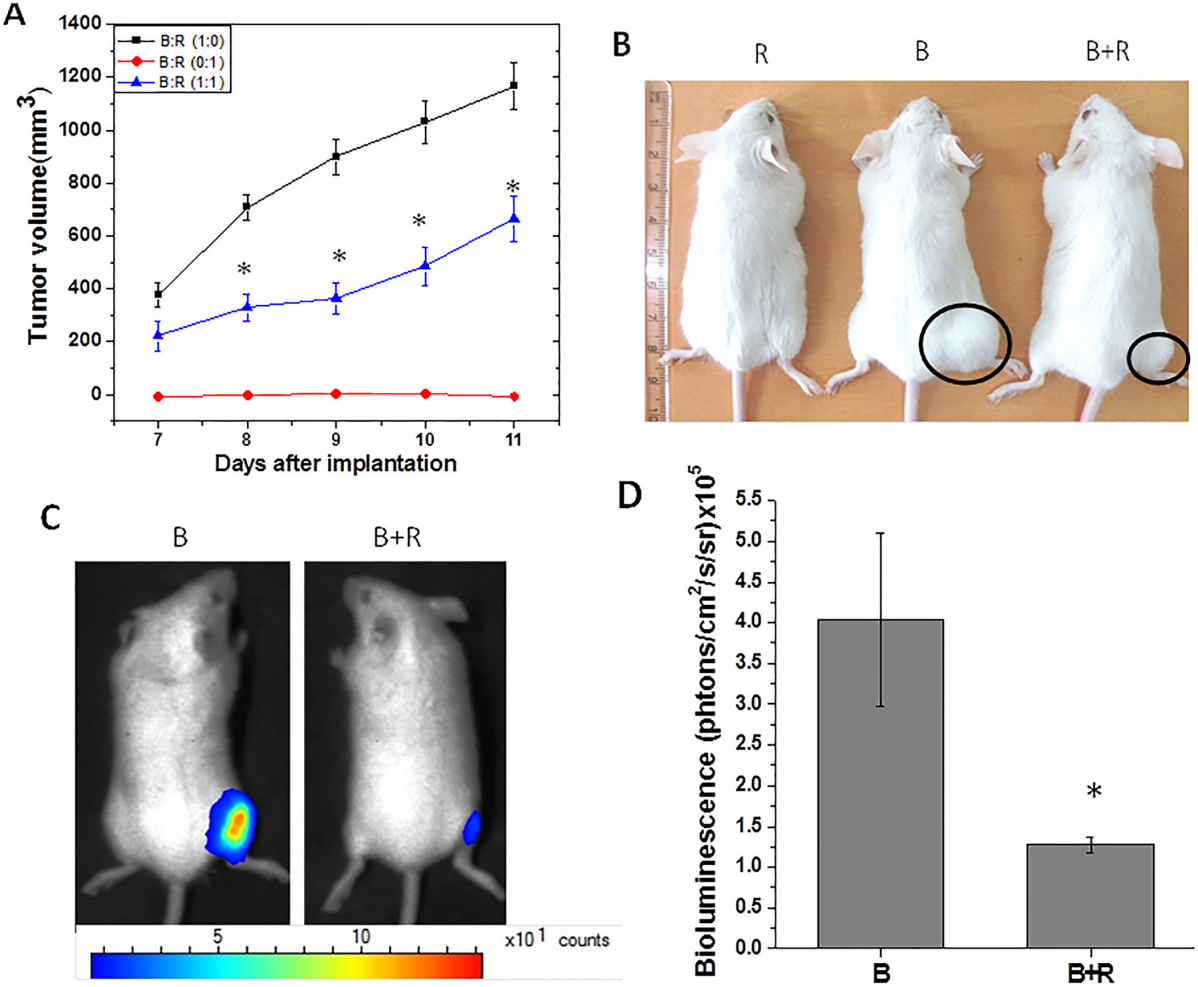
Irradiated tumor cells affect the growth of bystander tumor cells. Irradiated tumor cells mixed with bystander tumor cells were injected in mice. The tumor growth was monitored by tumor volume measurements (A) and images of representative mouse from each group are shown (B). B: bystander/unirradiated cells, R: irradiated cells. B: R (1:0): only bystander cells, B: R (0:1): only irradiated cells, B: R (1:1): bystander and irradiated cells in equal ratio. Data presented as mean ± SD (n = 10). *statistically significant than B: R (1:0) group at p<0.05. (C) Luc^-^WEHI 164 cells irradiated with 15 Gy were mixed with unirradiated (bystander) Luc^+^WEHI 164 cells in an equal ratio. The tumor formation by bystander cells was monitored by in vivo imaging as shown in representative images. (D) Quantitative representation of difference in bioluminescence signal. B: mice injected with bystander cells (luciferase positive) only, B+R: mice co-implanted with bystander cells (luciferase positive) and irradiated Luc^-^WEHI 164 cells. *statistically significant than B at p<0.05.

After the optimization experiments, effect of *ex vivo* irradiated tumor cells was studied on bystander tumor cells in terms of growth of tumor formed by these (bystander) tumor cells. In this experiment, unirradiated (bystander) cells were mixed (1:1) with irradiated cells (15 Gy) (referred to as ‘mixed cells’) and implanted in the animals. Tumor volume measurement of these animals revealed that as compared to the control group, animals implanted with mixed cells resulted in significantly smaller tumors and the difference in tumor volumes became more prominent at the later time points (Fig 1A and 1B). Compared to control tumors, in the mixed cells tumors, the % inhibition in the tumor volume was 60%, 72% and 54% at 8^th^, 9^th^ and 11^th^ day of tumor transplantation, respectively. The observed decrease in the growth rate of mixed cells tumors than that of control tumors suggest transfer of damaging signals from the irradiated tumor cells to bystander tumor cells. Further, evidence was also generated in the support of growth inhibitory bystander effect of irradiated tumor cells using *in vivo* imaging. For this, bystander (Luc^+^WEHI 164 cells) and irradiated (15 Gy; WEHI 164 cells) cells were co-implanted in mice. In this experiment, since only bystander cells will give the bioluminescence signal, the decreased growth of bystander cells (if any) in the presence of irradiated cells will be reflected in terms of decrease in bioluminescence signal. As anticipated, compared to bioluminescence signal obtained from the mice implanted only with Luc^+^WEHI 164 cells (4x10^5^±1x10^5^ photons/cm^2^/S/Sr), the mice implanted with mixture of cells (Luc^+^WEHI 164 cells and 15 Gy irradiated WEHI 164 cells) showed ~3 fold lower signal (1.25x10^5^±0.1x10^5^ photons/cm^2^/S/Sr). This confirmed that the growth inhibition observed in bystander tumor cells was due to the damaging signals from the irradiated tumor cells (Fig 1C and 1D). To rule out the possibility whether irradiated PBS itself hampers the tumor growth, only PBS was irradiated (15 Gy) and then mixed with control cells. This cell mixture was implanted in mice. The growth observed in these tumors was similar to that obtained with unirradiated PBS mixed with control cells (data not shown), suggesting irradiated media (PBS) itself does not affect the tumor growth. It may be argued that even though irradiated (15 Gy) cells could not form tumor when they are implanted alone (S2 Fig) but these cells may survive when they are co-implanted with bystander cells and thus contribute in the formation of mixed cells tumors. To counter the argument, irradiated (15 Gy) Luc^+^WEHI 164 cells were co-implanted with unirradiated WEHI 164 cells followed by measurement of bio-luminescence signal at different days after implantation. Results showed a decrease in signal intensity at day 5 (compared to day 3) but at longer time periods no bio-luminescence signal was detected. These results suggest that irradiated cells did not survive for more than 5 days after co-implantation with bystander cells and thus, not likely to contribute to the mixed cells tumors (S3 Fig).

### Histological changes in mixed cells tumors and control tumors

Histological changes were studied in mixed cells tumors and control tumors stained with hematoxylin and eosin. Parameters were analysed based on the size, shape, orientation and staining characteristics of cells. Mitotic index was calculated with the help of ratio of round to spindle shaped cells, as round cells indicate actively dividing cells whereas spindle shaped cells point out the quiescent cells. Similarly, cells arranged in different directions indicate anaplasia. Presence of cells with intensely stained nuclei is a characteristic of hyperchromatia indicating intact nuclear material/proliferating cells.

**Fig 2:**
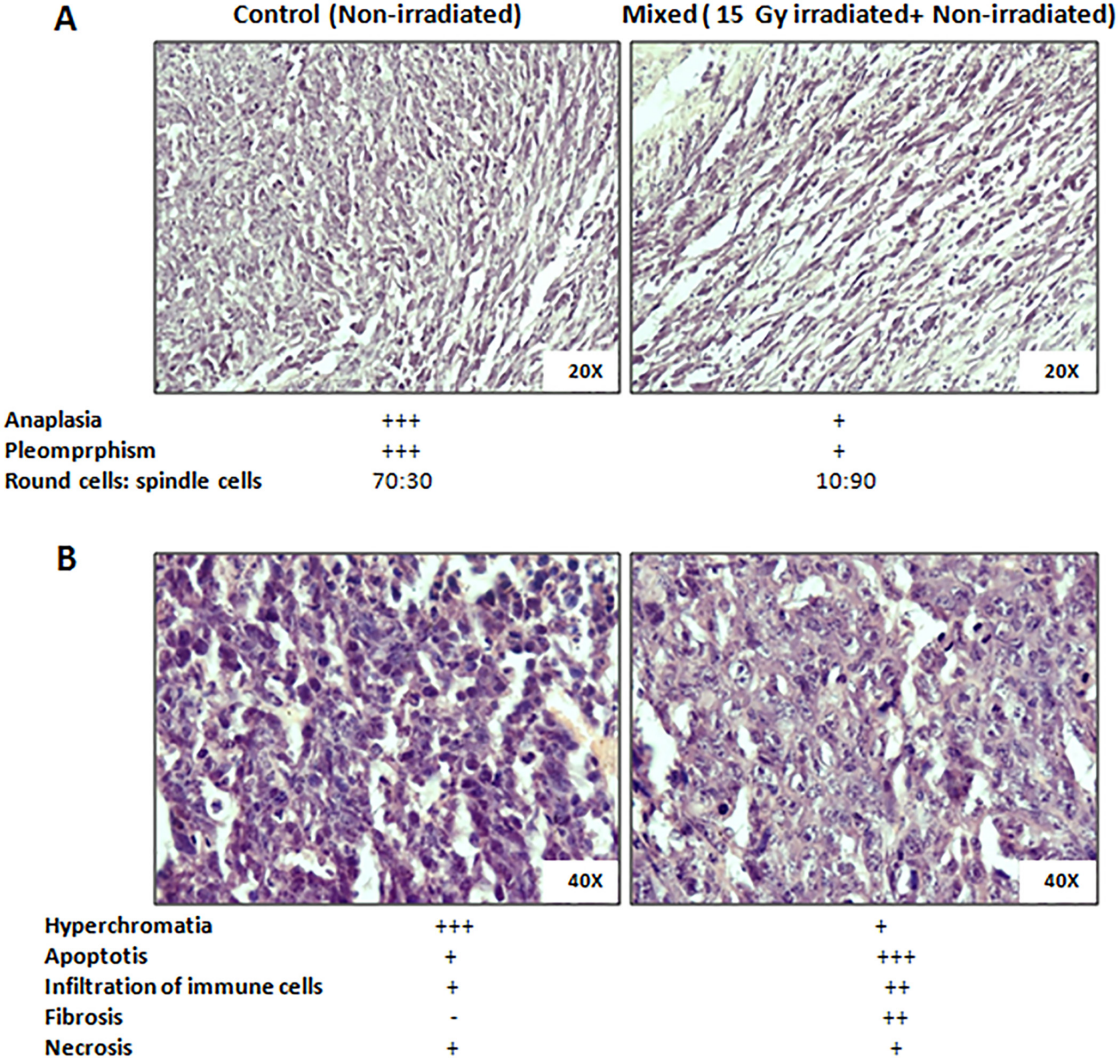
Histological analysis of tumor samples obtained from mice co-implanted with irradiated and bystander cells. Microscopic images at (A) 20X and (B) 40X. Results were expressed in terms of grades or scores ranging from 0 to +3 based on the severity/intensity, where -:absent, +: poor, ++:high, +++:severely high.

Gross analysis of sections of control tumors revealed pleiomorphic (having different shapes) and anaplastic (score +3) cells, the hallmark feature of poorly differentiated and aggressively growing tumor. Moreover, the ratio of round to spindle cells was found to be more than one (70:30) in the section of control tumors, indicating higher mitotic index in these tumors. On the contrary, the mixed cell tumors exhibited decreased pleiomorphism and anaplasia (score +1) with round: spindle cells ratio less than 1 (10:90), the characteristics of differentiated and slow growing (regressing/quiescent) tumor (Fig 2A). Further analysis of these results at higher magnification revealed a basal level of micronecrosis in the tumors from the both groups. However, compared to the control group, mixed cells tumors showed a significant increase in apoptotic cells (score 3+) and slight increase in the infiltration of polymorphonuclear leukocytes. Taken together these results suggest significant growth inhibitory pattern in the mixed cells tumors than the control tumors (Fig 2B).

### Role of apoptosis and senescence in decreased tumor growth caused by bystander signaling from the irradiated cells

**Fig 3:**
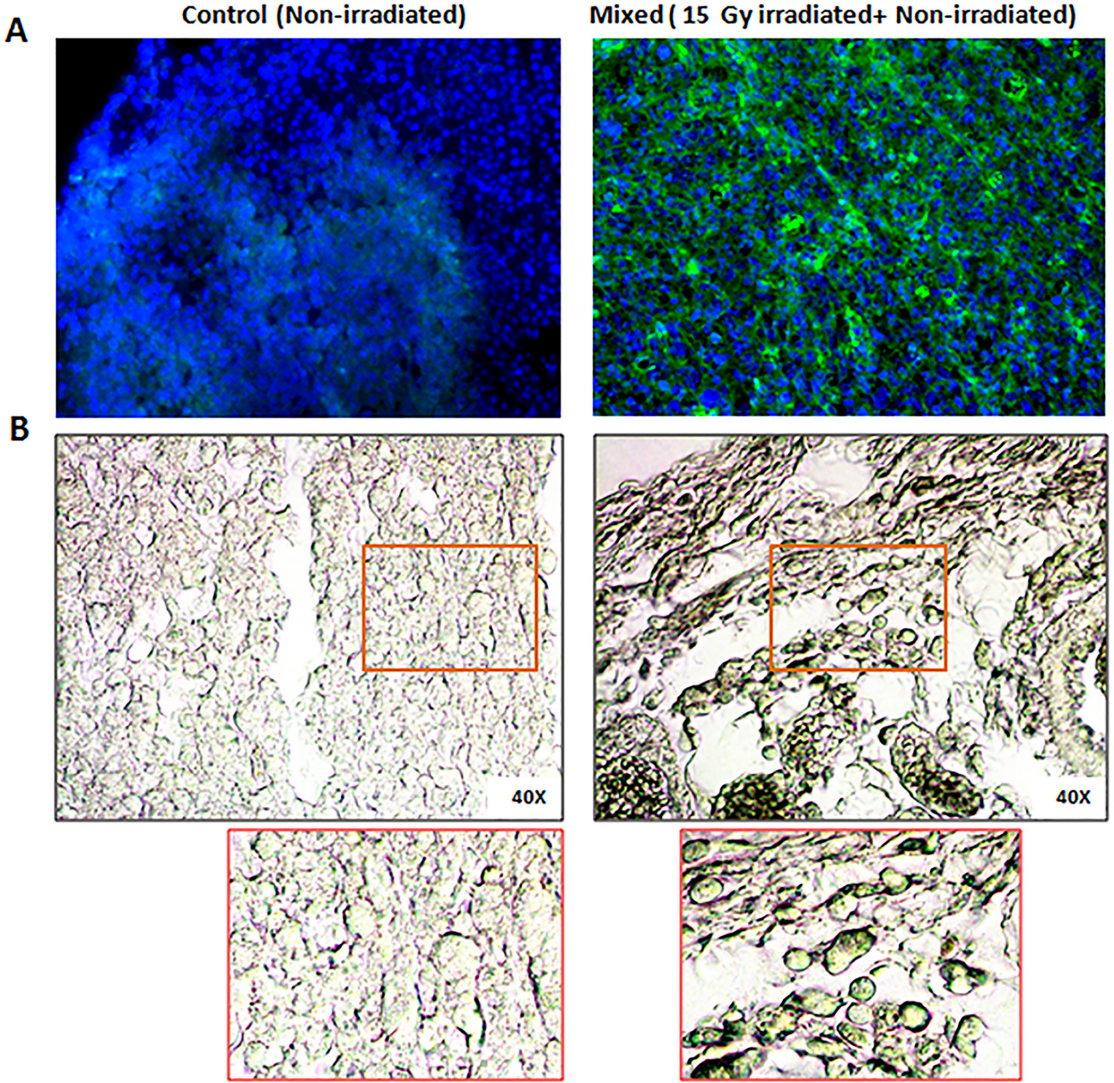
Apoptosis and senescence in tumor sections from control and mixed cells. (A) Sections of tumor tissues were subjected to TUNEL assay. Blue colour shows nuclei of cells stained with DAPI. Green foci show TUNEL positive regions. (B) Tumor sections processed for SA-β-gal assay. The part of image is shown below in the respective inset. Greenish blue color indicates active SA-β-galactosidase.

Tumor sections were processed for TUNEL assay to investigate the degree of apoptosis. Results showed remarkable increase in the fraction of apoptotic cells in the mixed cells tumors in contrast to that of control tumors (Fig 3A), which was consistent with histopathological observations (Fig 2A and 2B). The histological analysis of mixed cells tumors indicated senescence features, which were further confirmed by senescence associated β-galactosidase (SA-β-gal) activity assay in the tumor sections. Interestingly, results showed significantly higher SA-β-gal activity in the mixed cells tumors (than control tumors) and the observed enhanced senescence may be involved in tumor growth inhibition in this group of animals (Fig 3B).

### Effect of irradiated tumor cells on the magnitude of angiogenesis in mixed cells tumors

**Fig 4:**
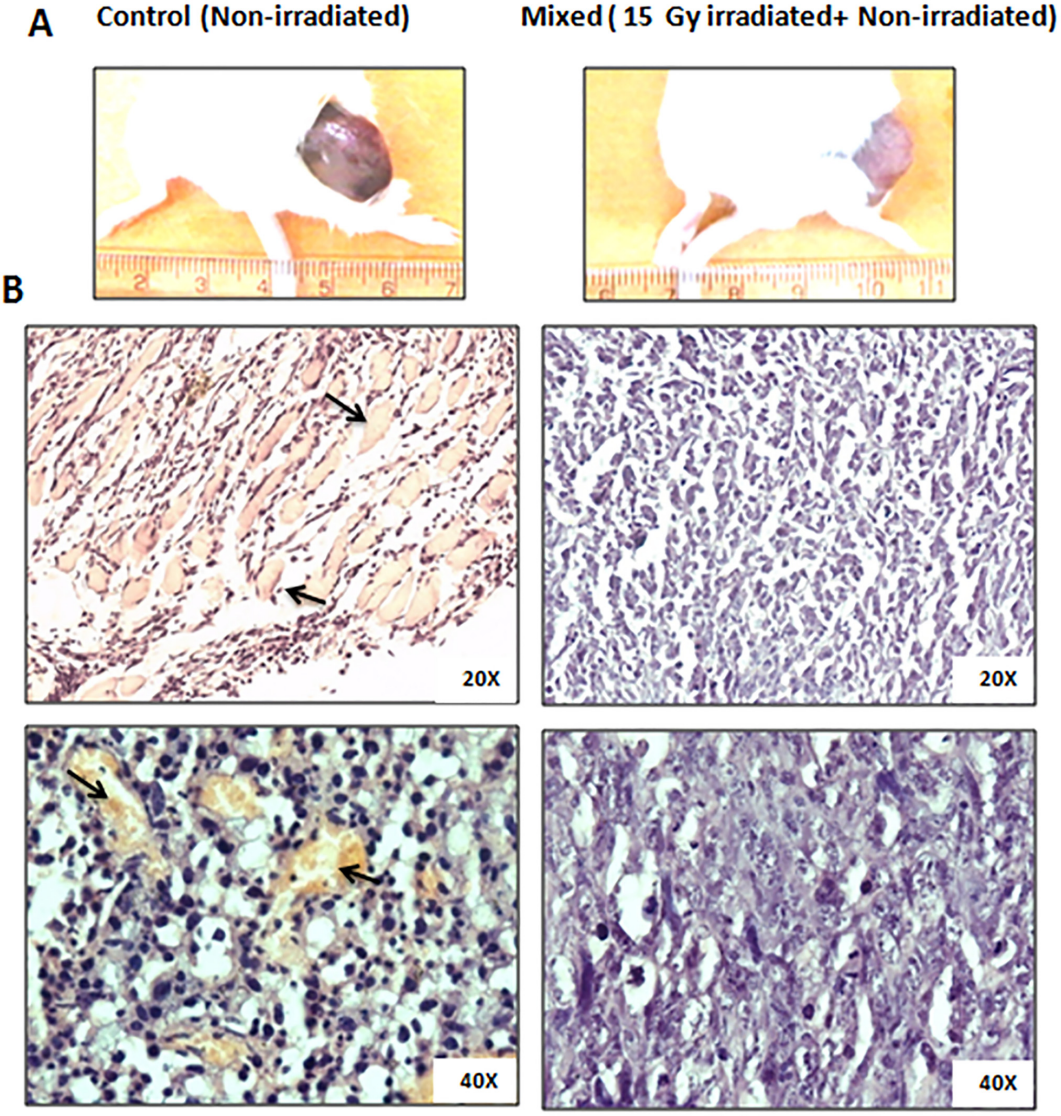
Gross and histological features of control tumors and mixed cells tumors. (A) Tumors obtained from co-implanting irradiated and bystander cells lack extensive vasculature unlike control tumors, which was visually evident. Scale shown in cm. (B) Microscopic histological analysis (40 and 60 X), wherein sections of tumor from control cells showed abundant capillaries (marked as arrows), which were barely seen in tumor sections from mixed cells.

**Fig 5:**
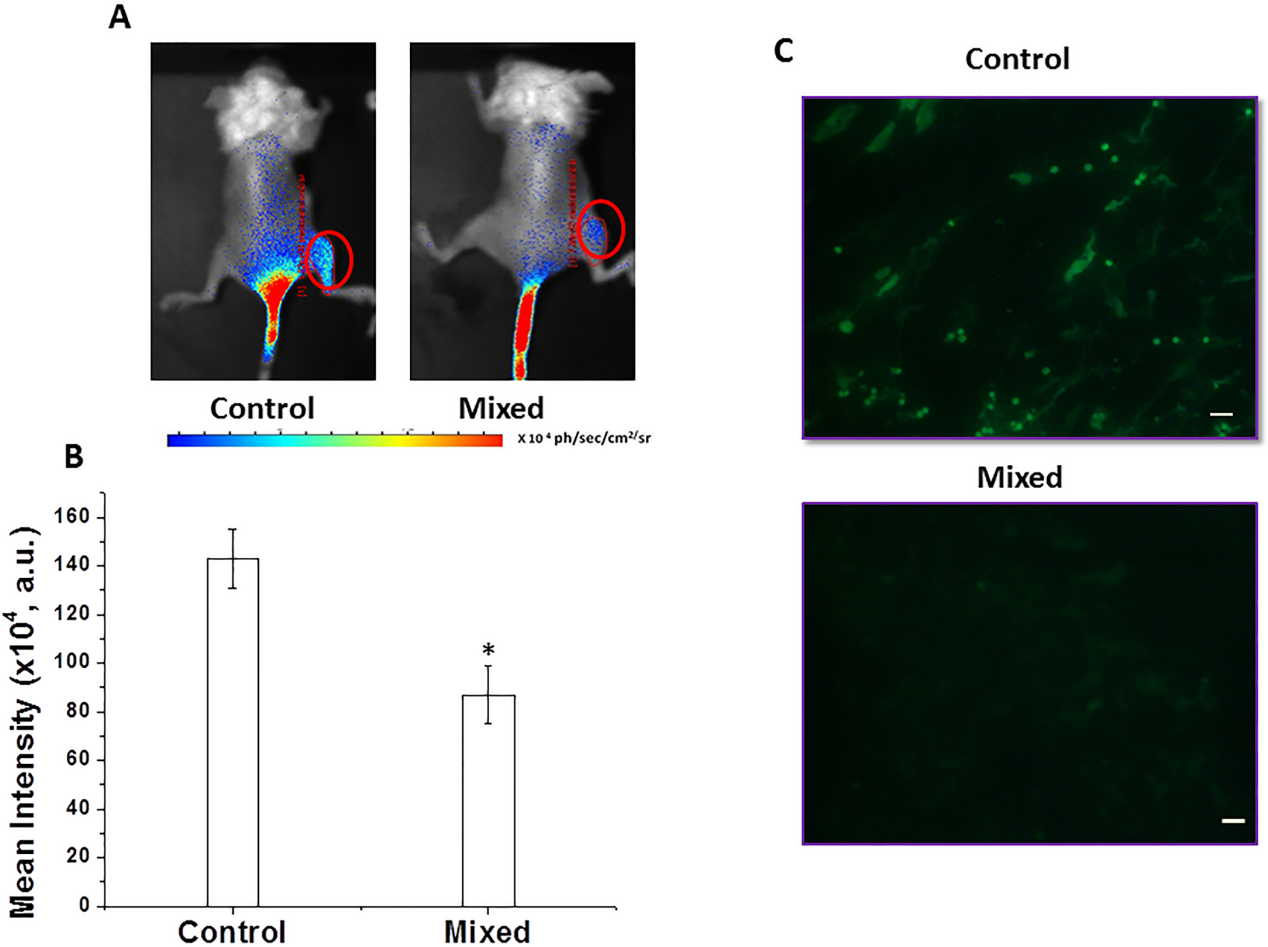
Effect of irradiated cells on angiogenesis in tumor formed out of bystander cells. (A) Tumor implanted with 1x10^6^ WEHI 164 cells (control) or tumor implanted with 1x10^6^ WEHI 164 unirradiated cells and 15 Gy irradiated 1x10^6^ WEHI 164 cells (mixed). At 11^th^ day of implantation, fluorescence imaging of animals were performed as mentioned in materials and methods. Representative images of animals from each group are shown. (B) Fluorescence intensity signal of animals (n = 6) was quantified. *significantly different than control at p<0.05. (C) Immuno-histochemistry of CD31 expression in tumor samples. Green areas indicate vasculature regions. Scale bar: 20 μm.

Gross visualization of tumors from dissected mice at 11^th^ day of implantation revealed prominent difference in the vasculature of mixed cells tumors than control tumors (Fig 4A). The control tumors were found to be bigger and dark red in colour with blood-vasculature rich areas (visible dark red patches). On the contrary, the mixed cells tumors were smaller in size, light pink in colour with fewer or no red patches (Fig 4A). The visual observation was further supported by histological analysis of tumor sections, which showed abundant blood capillaries in control tumors but not in mixed cells tumors (Fig 4B). Magnitude of angiogenesis in control tumors and mixed cells tumors were also assessed by fluorescence imaging of tumor bearing animals. For this, animals were injected with AngioSense dye followed by imaging after 24h of dye injection. Significantly lower accumulation of dye was observed in the mixed cells tumors than those of control tumors (Fig 5A). The quantitative analysis of imaging experiment showed ~40% decrease in fluorescence intensity (Fig 5B) in mixed cells tumors. Furthermore, change in angiogenesis was also validated using immuno-histochemistry of CD31 expression in the sections of control tumors and mixed cells tumors. Results showed that as compared to control tumors, the expression of CD31 was significantly lower in the mixed cells tumors. These results (Figs 4 and 5) suggest relatively hampered angiogenesis in the mixed cells tumors, which may be contributing in the slower tumor growth in this group of mice.

### Effect of soluble factors from the irradiated cells on the tumor growth of bystander cells

**Fig 6:**
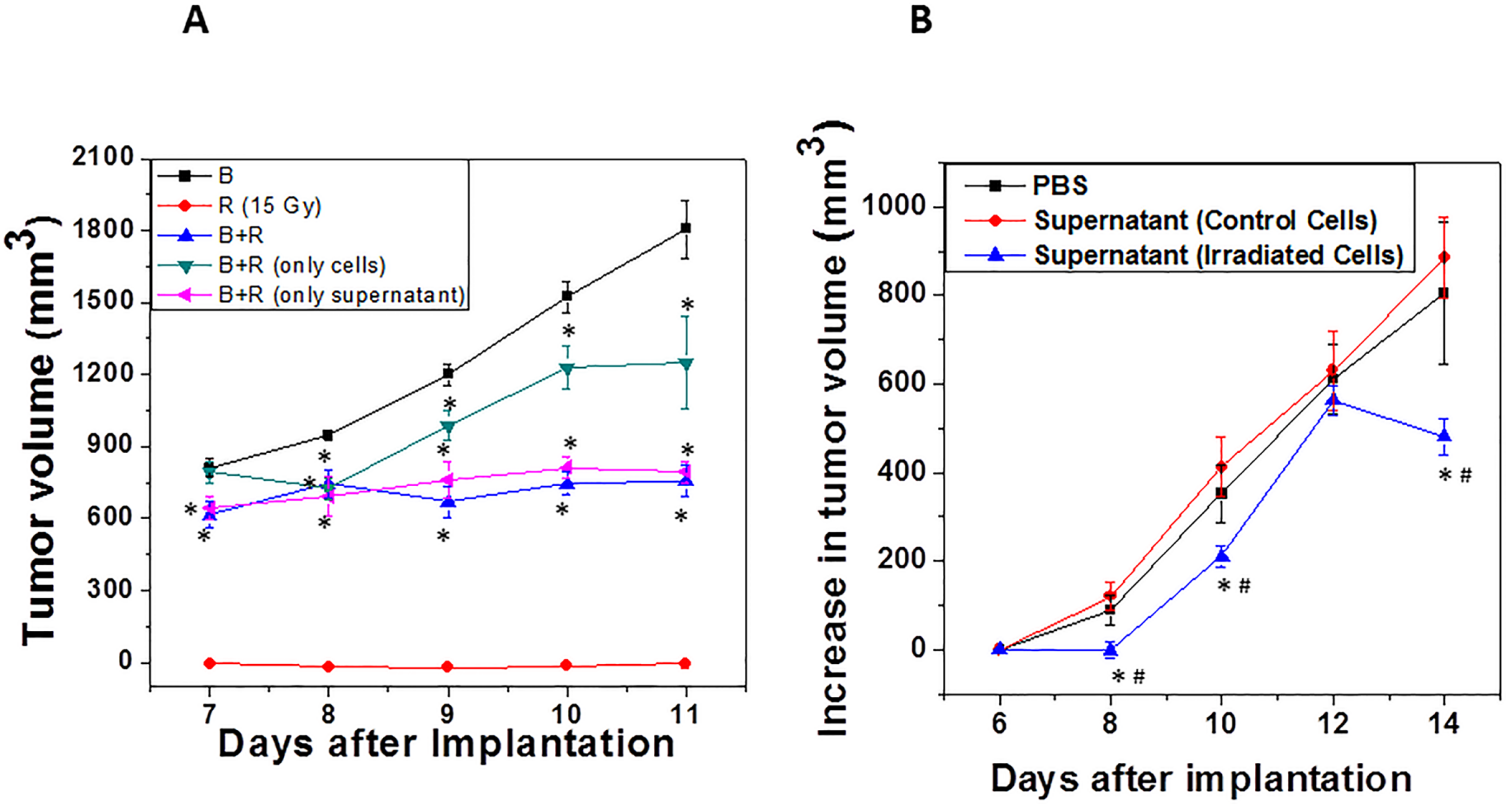
Effect of soluble factors secreted from irradiated tumor cells on the tumor growth of bystander cells. (A) Bystander cells were mixed either with the supernatant or the cell pellet obtained from irradiated cells and injected in hind legs of mice for tumor development. B: bystander cells (1x10^6^) R: irradiated cells (1x10^6^), B+R: mixture of bystander (1x10^6^) and irradiated cells (1x10^6^), B+R (only cells): Bystander cells (1x10^6^) mixed with irradiated cells (1x10^6^) pellet (1:1), B+R (only supernatant): bystander cells (1x10^6^) mixed with supernatant (50 μl) from (1x10^6^) irradiated cells. *statistically significant than B at p<0.05; (B) The mice were injected with unirradiated cells and allowed to develop a tumor to a palpable size (which took 6 days post-implantation). Once this size was achieved, supernatant from the control and irradiated cells (1x10^6^) was injected on 7^th^ and 9^th^ day after tumor implantation and tumor growth was measured. PBS: tumor injected with 100 μl PBS; supernatant (control cells): tumors injected with supernatant from the unirradiated cells; supernatant (irradiated cells): tumors injected with the supernatant from the 15 Gy irradiated cells. *statistically significant than PBS at p<0.05, #statistically significant than supernatant (control cells) at p<0.05;.n = 10.

To obtain the mixed cells tumors, the irradiated cells were used which contain two fractions i.e. the cells and the supernatant (with soluble factors). Hence, to study the relative contribution of these two fractions in the decreased growth observed in this group of animals following experiment was designed. Irradiated cells were centrifuged (15 Gy; 15 min post irradiation; 400 x g; 10 min; room temperature) to separate the cell pellet and the supernatant. The bystander cells were mixed either with the supernatant or with the cell pellet and these two cell mixtures were injected separately into the hind legs of mice to obtain the tumor. Subsequently tumor growth was monitored. Interestingly, compared to the control group, animals implanted with cell pellet (from irradiated cells) mixed with bystander cells showed significantly smaller tumor. However, in the case of animals injected with bystander cells along (mixed) with supernatant from the irradiated cells, the tumor volume was further lower and comparable to that of animals bearing tumors when whole cells (cells along with the supernatant) were mixed with bystander cells (Fig 6A). This result suggested that soluble factors from the irradiated cells (in the supernatant) are more effective in eliciting the observed damaging bystander effect than the irradiated cells themselves. Therefore, the protocol was further modified to test the therapeutic efficacy of the supernatant (from 15 Gy irradiated cells) on the tumor growth once the tumor has been formed. For this, using unirradiated tumor cells, mice were allowed to develop tumor of a palpable size (which took 7 days post-implantation). Once this size was achieved, supernatant from the irradiated cells (100 μl) was injected intra-tumorally (twice with a gap of two days between two injections i.e. on 7^th^ and 9^th^ day after implantation) and tumor growth was monitored. Results showed that compared to control group (i.e. supernatant from the control cells), the increase in tumor volume was significantly lower in the animals injected with supernatant from the irradiated cells (Fig 6B).

### Cytokine profiling and proteomics analysis of supernatant from the irradiated tumor cells

**Fig 7:**
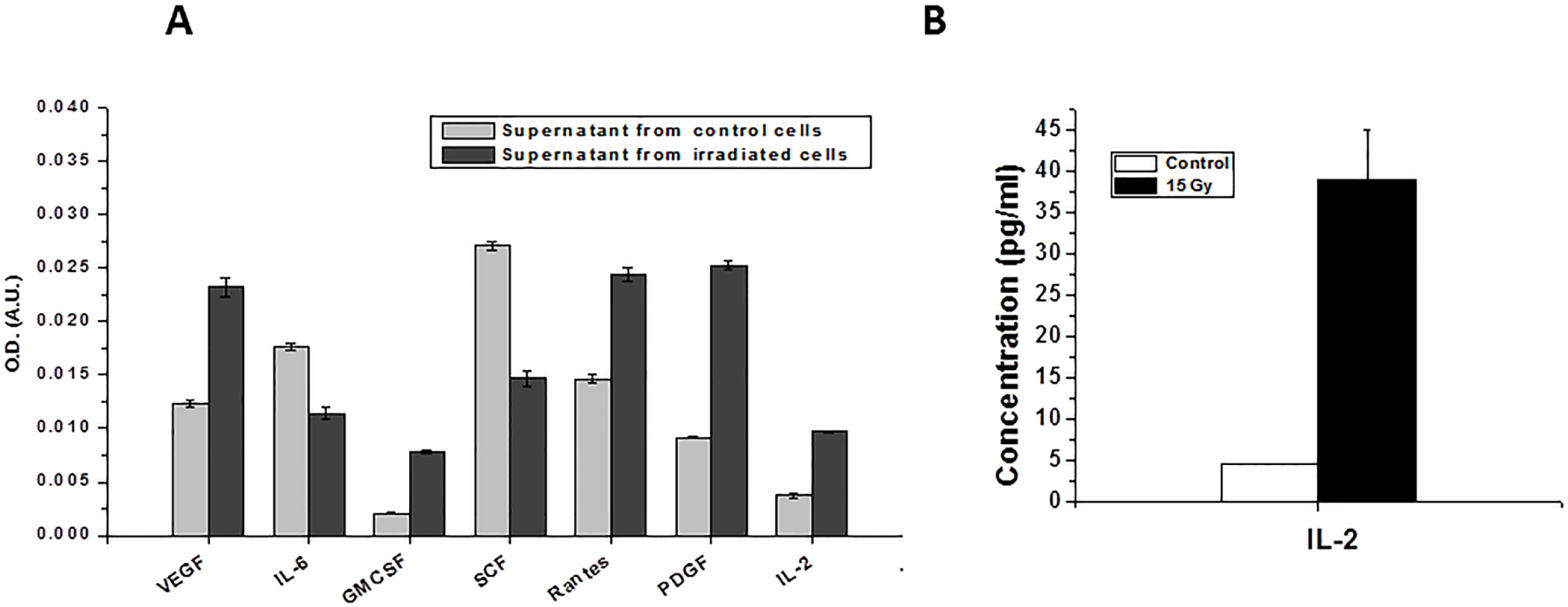
Characterization of supernatant from irradiated WEHI 164 cells. **(A)** Supernatant from the control and irradiated cells was subjected to ELISA panel for quantitative analysis of cytokines, chemokines and growth factors. (B) Measurement of concentration of IL-2 by ELISA in the supernatants from control and irradiated cells.

The tumor growth inhibitory bystander response exerted by irradiated WEHI 164 cells was found to be pronouncedly mediated by soluble fraction in the supernatant obtained from the irradiated cells. Hence, the supernatant was further analysed to identify the putative mediators for the observed tumor growth inhibitory effect. For this, supernatant obtained from the irradiated cells was subjected to an ELISA array, which included a panel of cytotoxic, pro-apoptotic and pro-inflammatory cytokines, chemokines and growth factors (namely TNF-α, IGF, VEGF, IL-6, FGFb, IFN-γ, EGF, Leptin, IL-1-α, IL-1-β, GCSF, GMCSF, MCP-1, MIP-α, SCF, Rantes, PDGF, b-NGF, IL-17A, IL-2, IL-4, IL-10 and Resistin). Results showed that compared to the supernatant from the control cells, the supernatant from irradiated cells showed significantly higher level of VEGF, GMCSF, PDGF and Rantes (Fig 7A). On the other hand, levels of SCF and IL-6 were found to be decreased significantly in the supernatant obtained from the irradiated cells. Other cytokines in array panel did not show any significant change in the supernatant from irradiated cells compared to that in the control cells. These results were further validated by the measurement of concentration of representative cytokine IL-2 by ELISA assay. IL-2 was found to be ~8 fold higher in the supernatant obtained from irradiated tumor cells than the supernatant from control cells (Fig 7B).

**Fig 8:**
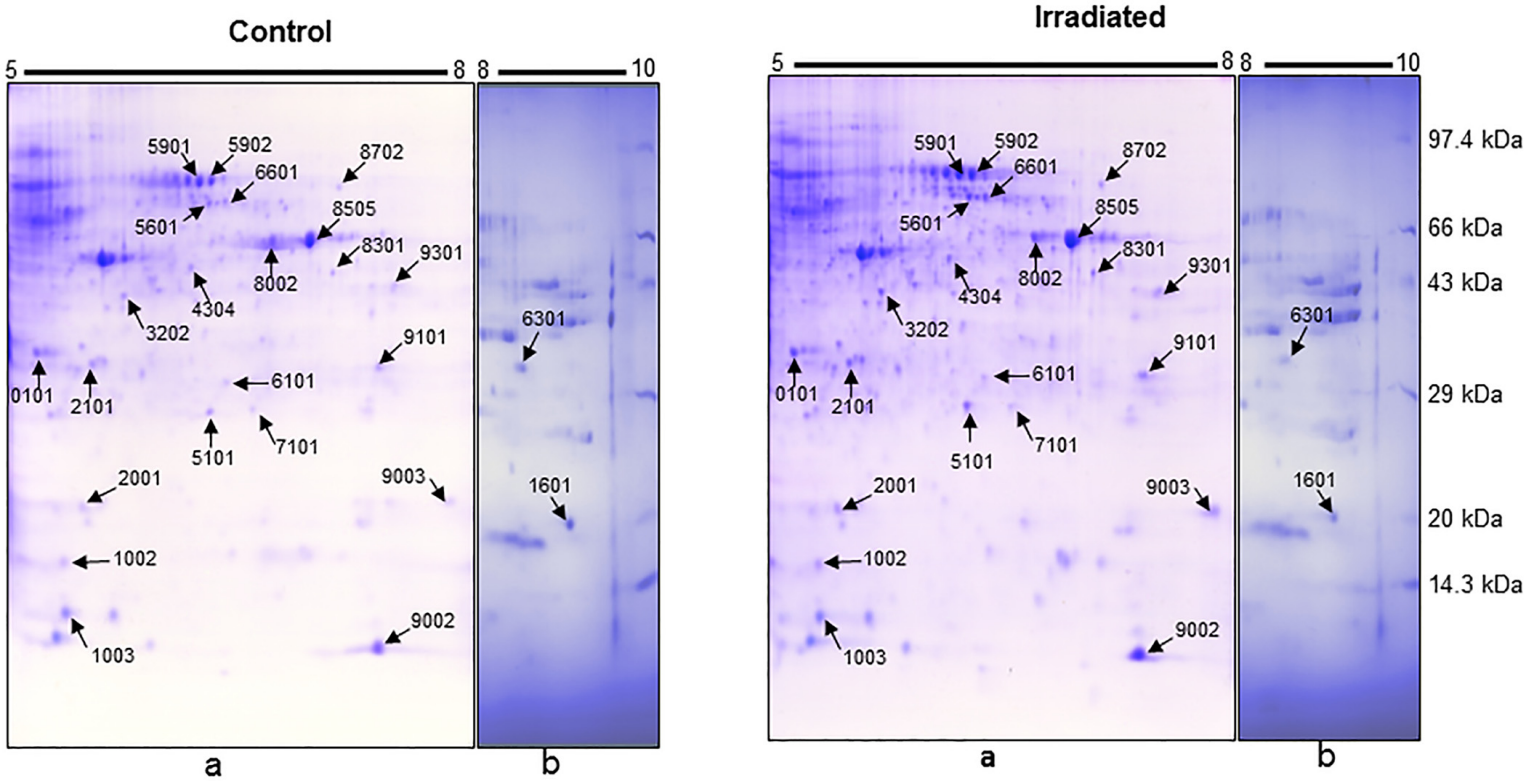
Representative 2DE images of total proteins from control and irradiated (15 Gy) cell supernatants. (a) Image of proteins resolved on pH 5–8 IPG strip (b) image showing pH 8–10 region of pH 3–10 IPG strip. The positions of differentially expressed proteins are marked with arrows and labelled with Standard Spot Number (SSP#).

To further characterize the protein profile secreted from the irradiated tumor cells, proteomic analysis of the supernatant was carried out using 2-D electrophoresis A representative distribution of proteins in the supernatants of unirradiated and irradiated WEHI 164 cells is shown in Fig 8. The proteins were resolved first on broad pH range IPG strips (pH 3–10) and then on narrow range IPG strips (pH 5–8). The 2D proteome spot pattern analysis using PD Quest program detected an average of 44±4 spots on pH 3–10 IPG strip and 121±2 spots on pH 5–8 IPG strip. Of these, a total of 24 protein spots were found to be differentially modulated (≥1.5-fold) between the supernatant obtained from the control and irradiated cells (21 over-expressed and 3 under expressed) (Fig 8). All the 24 proteins were identified with MALDI-TOF-MS and confirmed with MS-MS (Table 1). The major proteins that were over-expressed in the irradiated supernatant included cytoskeletal protein like actin (cytoplasmic 1/2), proteins involved in cellular metabolic processes (alpha-enolase, phospho glycerate mutase-1, nucleoside diphosphate kinase B, adenine phosphorybosyl transferase), antioxidant protein (peroxiredoxin-6), chaperone protein (heat shock 71kDa cognate), stress-induced phosphoprotein 1 protein CIP2A (controls cell growth and autophagy), galectin-1 (protein involved in immune response, inflammation, allergic reaction and host pathogen interaction), serine/threonine-protein phosphatase PP1-gamma catalytic subunit (phosphoprotein having phosphatase activity), angiopoetin-2 (regulator of angiogenesis), ubiquitin-60S ribosal protein L40 (involved in proteosome mediated protein degradation) and annexin A1 (calcium/phospholipid-binding protein involved in exocytosis). The under expressed proteins were protein S100-A4 (involved in cell cycle progression, differentiation and inflammation), mixture of annexin A2 (play role in exocytosis, endocytosis and membrane trafficking) and lactate dehydrogenase (carbohydrate metabolism) and cofilin (actin binding protein) (Table 1).

**Table 1:** List of differentially expressed proteins from the supernatant of tumor cells identified by MALDI TOF Mass Spectrometry. Represented fold change is with respect to supernatant from unirradiated control cells. The symbol (-) depicts down regulation. The proteins are listed with #SSP numbers as labeled on Fig 8.

Spot No.	Protein Name	SWISS PROT Accession Number	Mascot Score	Sequence coverage (%)	No. of peptide matches	Mr (KDa) / pI		Fold Change
						Theoretical	Measured	
0101	Actin, cytoplasmic 1/2	P60710/ P63260	81	30.4	8	42.0/5.2	30.0/5.1	1.69±0.07
1002	Galectin-1	P16045	118	61.5	8	15.2/5.2	14.0/5.2	2.54±0.62
1003	Protein S100-A4	P07091	76	34.7	6	12.0/5.1	11.0/5.2	-1.50±0.18
1601	Cofilin-1	P18760	77	47.0	7	19.0/9.1	19.0/9.3	- 2.0 ± 0.06
2001	Actin, cytoplasmic 1/2	P60710/ P63260	58	18.1	5	42.0/5.2	17.0/5.32	2.25±0.26
2101	Actin, cytoplasmic 1/2 (120PPM)	P60710/ P63260	68	29.3	9	42.0/5.2	29.3/5.4	1.63±0.06
3202	Alpha-enolase	P17182	110	32.3	12	48.0/6.4	36.0/5.5	1.68±0.12
4304	Actin Cytoplasmic 1/2	P60710/ P63260	87	40.5	10	42.0/5.2	41.0 /5.8	1.89±0.09
5101	Heat shock cognate 71kDa protein	P63017	89	23.7	16	71.0/5.2	25.0/5.9	2.68±0.26
5601	Protein CIP2A	Q8BWY9	55	10.3	11	103/5.9	57.0/5.9	2.14±0.09
5901	Serine/threonine-protein phosphatase PP1-gamma catalytic subunit	P63087	56	29.4	6	37.7/6.1	67.0/5.8	2.13±0.11
5902	Angiopoietin-2	O35608	59	18.8	8	57.0/5.3	67.0/5.9	2.15±0.17
6101	Peroxiredoxin-6	O08709	125	45.5	10	25.0/5.6	27.0/6.0	1.90±0.22
6301	Annexin A2	P07356	116	41.6	15	39.0/8.5	39.0/8.5	-1.70± 0.09
	L-lactate dehydrogenase A chain	P06151	107	53.6	15	37.0/8.8	39.0/8.5	
6601	Serine/threonine-protein phosphatase PP1-gamma catalytic subunit	P63087	62	19.2	5	37.7/6.1	57.0/6.0	2.24±0.23
7101	Adenine phosphoribosyl transferase	P08030	113	38.9	7	19.9/6.4	25.0/6.1	1.61±0.09
8002	Alpha-enolase	P17182	163	54.1	22	48.0/6.4	43.7/6.2	2.15±0.20
8301	Alpha-enolase	P17182	108	31.6	12	48.0/6.4	39.0/6.6	1.75±0.11
8505	Alpha-enolase	P17182	169	48.4	21	48.0/6.4	43.0/6.4	2.65±0.55
8702	Stress-induced phosphoprotein 1	Q60864	143	38.9	22	64.0/6.4	64.0/6.6	2.25±0.05
9002	Ubiquitin-60S ribosomal protein L40/ Ubiquitin-40S ribosomal protein S27a/Polyubiquitin-B/C	P62984	86	46.1	7	15.0/10.7	10.0/7.0	2.23±0.14
9003	Nucleoside diphosphate kinase B	Q01768	112	52.0	7	17.5/7.8	17.5/7.8	6.06±0.5
9101	Phosphoglycerate mutase 1		154	61.0	11	29.0/6.8	29.0/7.0	2.19±0.08
9301	Annexin A1	P10107	81	31.5	9	39.0/7.7	37.0/7.1	2.39±0.05

## Discussion

The phenomenon of radiation induced bystander effect has been associated with low as well as high doses of radiation. At low doses of radiation, contribution of RIBE has been well investigated as a modifier of the cellular response. At high doses of radiation, though the direct effects of radiation predominate, the contribution of bystander effect in the systemic radiobiological response cannot be underestimated. Especially during cancer radiotherapy, signalling from the high dose irradiated tumor cells may contribute towards enhanced tumor cell killing due to bystander effects [22, 23]. However, till date, there are only a few reports that address this issue in the context of cancer radiotherapy. Validation of such effects may have prominent impact on radiotherapy planning. Hence, in the present study, we have attempted to validate the existence and effect of radiation-induced bystander signalling in a tumor model. To achieve this, *ex vivo* irradiated cells were mixed with unirradiated cells and implanted in mice legs to form tumor. Results showed that the lethally irradiated cells hamper the overall growth of tumor. The tumor growth retarding effect may be ascribed to multiple effects exerted by irradiated cells on bystander cells. At one side, these effects were found to induce senescence and apoptosis in bystander cells, on the other hand, under the influence of bystander signalling the tumor angiogenesis was inhibited at the tissue level. These results are consistent with reports showing occurrence of apoptosis in cells bystander to irradiated cells in other experimental models [8, 23, 24]. As compared to apoptosis which is considered to be a faster radiation induced cell death process, senescence is relatively slower and well controlled phenomenon contributing to tumor suppression [25]. Senescence processes can cause tumor suppression through arrest of pre-transformed cells proliferation and slow acquisition of additional oncogenic events in these cells. Moreover, the senescence events may also contribute in the inhibition of tumor growth by clearance of pre-transformed cells [25]. In the present work, this line of interpretation was supported by the identification of extracellular soluble factors secreted by irradiated cells as molecular mediators of the observed effect. The putative mediators of tumor inhibitory effect of supernatant from the irradiated cells seem to involve radiation-induced upregulation of VEGF, PDGF, GMCSF and IL-2 and downregulation of IL-6 and SCF. These factors (VEGF, PDGF and GMCSF) are basically growth factors and are required for the cell survival and proliferation under normal conditions [26]. Their increased level under stress conditions may support the recovery of radiation damaged cells. However, secretion of these factors from the irradiated cells at higher level may be cytotoxic to bystander cells. Moreover, in response to radiation, tumor cells secrete pro-angiogenic cytokines like VEGF and PDGF to compensate the damaged blood vessels for continued supply of blood to the tumor mass. Hence, the over-expression of PDGF is often related with fibrotic development. PDGF may also exert pro-fibrotic effect through its mitotic and chemotactic stimulations, which get further supported by other cytokines like TGF-β, TNF, IL-1 (secreted from endothelial cells and macrophages) that promote proliferation of fibroblasts [27]. This leads to excessive deposition of extracellular matrix and formation of irreversible fibrotic regions. This is in accordance with present findings wherein mixed cells tumors showed significant fibrosis/senescence, which may be associated with increased level of PDGF in the supernatant from the irradiated tumor cells. Interleukin-2 (IL-2) is a cytokine that is essential for lymphocytic survival and function [28]. IL-2 is known to exert anti-proliferative and pro-apoptotic effects on kidney cancer cells [29]. In agreement with this report, our results showed increased secretion of IL-2 from irradiated cells and thus increased apoptosis and slower proliferation in bystander cells. IL-6 and SCF cytokines are required for sustained survival and proliferation of different cell types [30, 31]. The decreased secretion of IL-6 in the supernatant of irradiated cells supernatant may explain the growth inhibition in bystander tumor cells.

Supernatant obtained from the lethally irradiated cells was found to have more pronounced tumor inhibitory effect than cellular fraction. The release of these soluble factors from the irradiated tumor cells seems to follow a fast kinetics since the supernatant was collected at 15 min post-irradiation. Results also showed ability of these soluble factors in the supernatant to slow down the growth of tumor even when they were administered once tumor appeared, suggesting therapeutic potential of these factors.

Proteomic analysis of the supernatants from irradiated cells revealed differential regulation for an array of proteins. The identified proteins could be subdivided into several groups based on their general biological functions such as energy metabolism, inflammation, apoptosis, cell-architecture and cellular stress. Upregulation of stress-induced and energy metabolism proteins has been associated with radiation stress and bystander effect [32]. The major proteins which showed more than 1.5 fold higher expression in the supernatant from the irradiated cells included nucleoside diphosphate kinase B, heat shock cognate, annexin A1, angiopoietin-2, actin (cytoplasmic 1/2), stress induced phosphoprotein 1 etc. Serine/threonine protein phosphatases antagonise the function of kinases and hence regulate many cellular pathways involved in cell proliferation and apoptosis [33]. Similarly, actins are a group of cell architecture proteins that are responsible for determining the cell shape, motility and invasion. Radiation induced changes in cytoskeletal proteins have been reported earlier [21]. On the other hand, cofilins are group of actin binding proteins that disassemble actin filaments. The increased level of actin and decreased level of cofilin-1 in the supernatant from the irradiated tumor cells appear to be associated with rearrangement of cellular cytoskeleton after a high dose of radiation [34]. In addition, irradiated cells also showed elevated secretion of galectin-1. Galectins are a family of beta-galactosidase binding proteins implicated in cell-cell and cell-matrix interaction. Galectin-1 may act as an autocrine negative growth factor that may regulate cell proliferation [35]. Like other members of family, galectin-1 is secreted into extracellular space, which is known to interact with various binding partners like integrins α5β1 and ganglioside GM1, thus resulting in inhibition of cell growth and thereby senescence in various cancer types like hepatocarcinoma, melanoma, breast and ovarian carcinoma [36, 37]. These results are in agreement with growth inhibitory effect of the supernatant from irradiated tumor cells containing higher level of galectin-1, however, the mechanism of its secretion and cytotoxic effect still needs to be studied. The protein S100A4 is known to regulate cell cycle progression, intercellular adhesion, invasion and metastatic properties of the cancer cells. The S100A4 protein is known to sequester and disable the p53 suppressor protein and thus affect the cell cycle transitions [38].

The anti-angiogenic effect of irradiated cells in mixed tumors (Fig 3) may be attributed to increased secretion of angiopoietin-2. Angiopoietins are a group of vascular growth factors that play an important role in embryonic and post-natal angiogenesis. Angiopoietin-2 (ANG-2) is known to differentially regulate the tumor angiogenesis. Though, in some cases, ANG-2 is known to promote angiogenesis, however, under certain conditions, it binds with endothelial-specific receptor tyrosine kinase 2 (TIE2) and act as a negative regulator of ANG-1/TIE2 signalling during angiogenesis through controlling the responsiveness of endothelial cells to exogenous cytokines [39]. It is quite possible that the elevated level of ANG-2 may inhibit angiogenesis observed in the mixed cells tumors through poor response of secreted cytokines to endothelial cells. Moreover, observed RIBE is also accompanied with infiltration of immune cells (Fig 4B); hence the role of inflammatory response in overall tumor growth retardation can’t be denied. This argument is supported by observed downregulation of annexin A2 and upregulation of annexin A1. Annexins are inhibitors of phospholipase A2, which are usually found in cells. However, some isoforms of annexins (A1, A2 and A5) are also known to be secreted out [40]. Increased expression of annexins is one of the mechanism by which glucocorticoids (cortisols) inhibit inflammation [41, 42]. Therefore, an upregulation of annexin A1 as seen in proteomic analysis may be involved in inhibition of neutrophil extravasation [43] and minimization of inflammatory response in the damaged tissue (anti-inflammatory response) [44]. On the other hand, downregulation of annexin A2 has been shown to result in excess deposition of fibrous material in tissue [45], which might explain higher fibrosis in mixed cells tumors (Fig 4B). It may be pertinent to mention here that even though only a few secreted proteins can be directly/indirectly implicated as putative mediator(s) in observed RIBE, these results provided an opportunity to explore their hitherto unknown role(s) in context of bystander and cancer radiotherapy.

In summary, the lethally irradiated tumor cells were found to slow down the growth of unirradiated tumor cells by inducing apoptosis and senescence. Moreover, these growth inhibitory effects were found to be mediated by soluble factors secreted from the irradiated cells. Such tumor cell-irradiation induced damaging bystander effects may have significant implications in the context of cancer radiotherapy.

## Supporting Information

S1 Fig Schematic of experimental approach for RIBE study in mouse fibrosarcoma tumor model. (TIF)

S2 Fig Determination of non-tumorigenic radiation dose. (A) Tumor volume measurement (B) image of representative mouse from each group at day 11.
(TIF)

S3 Fig Signal Intensity of irradiated tumor cells when mixed with bystander tumor cells in mice. Unirradiated (bystander; Luc^-^) and 15 Gy (irradiated; Luc^+^) were mixed at 1:1 and implanted to mice. Signal intensity was measured at day 3, 5 and 7 after implantation in mice (n = 6) using Imaging system as mentioned in materials and methods. At day 7 and longer period (day 9, 11 and 13), no signal was observed. a.u.: arbitrary unit. *statistically significant than B at p<0.05.
(TIF)
