# Supplementary figures and images for: Molecular Understanding of Growth Inhibitory Effect from Irradiated to Bystander Tumor Cells in Mouse Fibrosarcoma Tumor Model
Source: PLoS One. 2016 Aug 25;11(8):e0161662. doi: 10.1371/journal.pone.0161662 (PMC4999205; doi:10.1371/journal.pone.0161662)

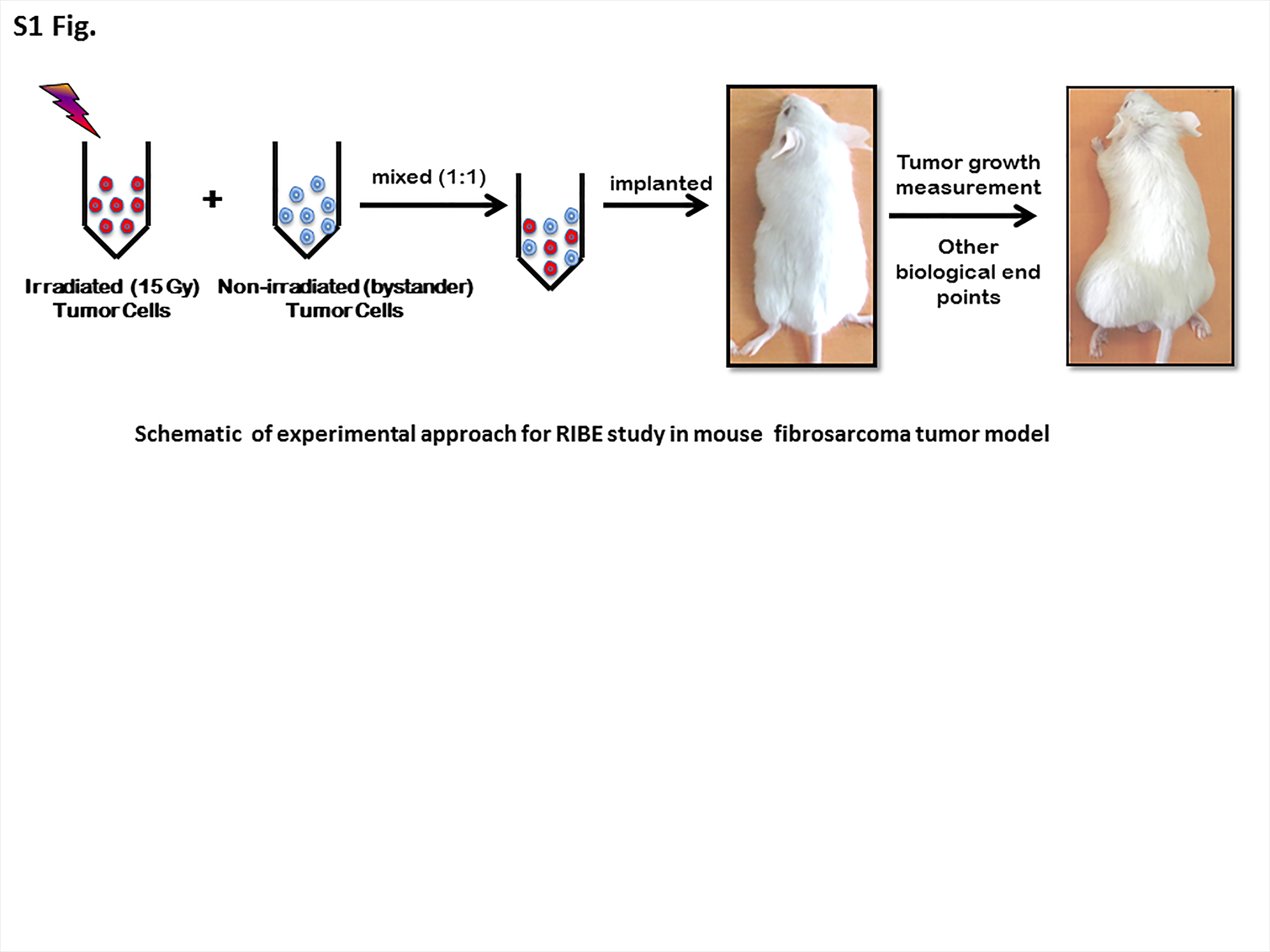

Supplement: S1 Fig — (TIF) [file pone.0161662.s001.tif]

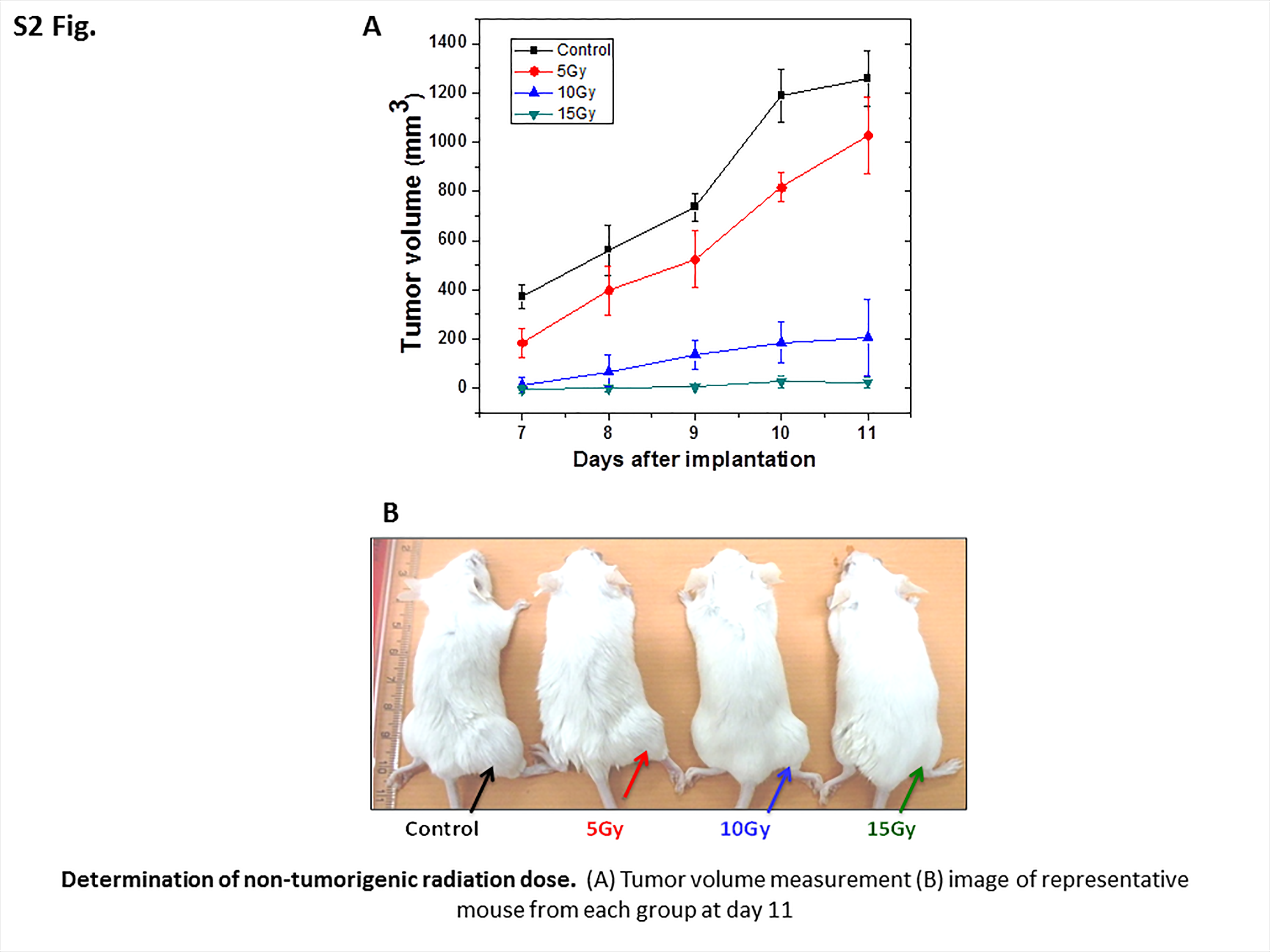

Supplement: S2 Fig — (A) Tumor volume measurement (B) image of representative mouse from each group at day 11. (TIF) [file pone.0161662.s002.tif]

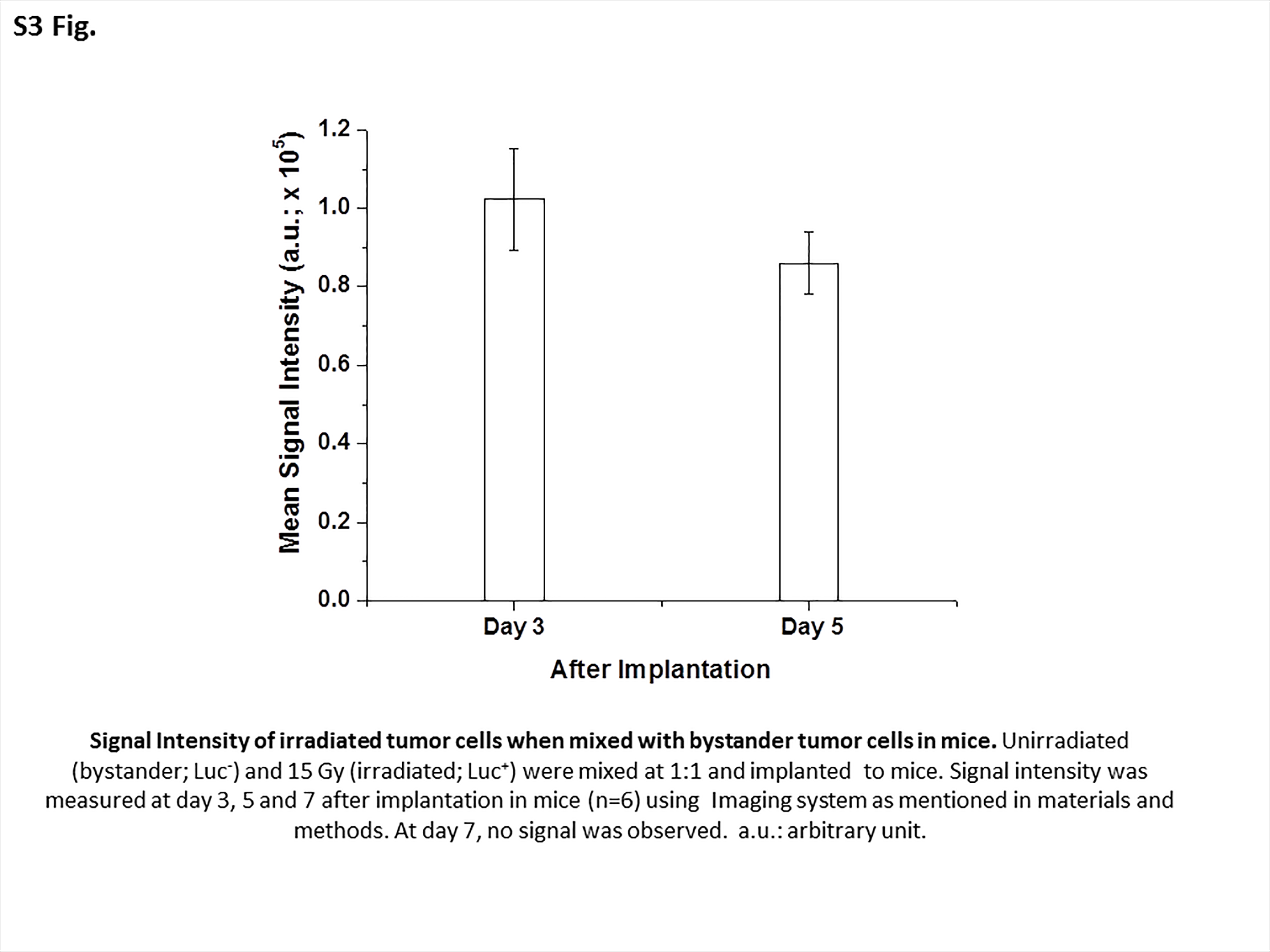

Supplement: S3 Fig — Unirradiated (bystander; Luc-) and 15 Gy (irradiated; Luc+) were mixed at 1:1 and implanted to mice. Signal intensity was measured at day 3, 5 and 7 after implantation in mice (n = 6) using Imaging system as mentioned in materials and methods. At day 7 and longer period (day 9, 11 and 13), no signal was observed. a.u.: arbitrary unit. *statistically significant than B at p<0.05. (TIF) [file pone.0161662.s003.TIF]
